# Supplementary material for: Targeting Wnt/β-Catenin Signaling Exacerbates Ferroptosis and Increases the Efficacy of Melanoma Immunotherapy via the Regulation of MITF
Source: Cells. 2022 Nov 12;11(22):3580. doi: 10.3390/cells11223580 (PMC9688625; doi:10.3390/cells11223580)
Supplement: Supplementary file 1 [file cells-11-03580-s001.zip › Cells_Supplementary Material.pdf]

Supplementary Material

1. Supplementary Tables

1.1 Supplementary Table S1: Sequence information for primers.

Supplementary Table S1. Sequence information for primers (5′ to 3′).

Axin2	Forward Primer: CAAACTTTCGCCAACCGTGGTTG<br>Reverse Primer: GGTGCAAAGACATAGCCAGAACC
Cyclin D1	Forward Primer: CCTCTGTGCCACAGATG<br>Reverse primer: GGGTCACACTTGATCACTC
Myc	Forward Primer: CCTGGTGCTCCATGAGGAGAC<br>Reverse Primer: CAGACTCTGACCTTTTGCCAGG
MITF	Forward Primer: GGCTTGATGGATCCTGCTTTGC<br>Reverse Primer: GAAGGTTGGCTGGACAGGAGTT
PGC1α	Forward Primer: GGCAGTAGATCCTCTTCAAGATC<br>Reverse Primer: TCACACGGCGCTCTTCAATTG
SCD1	Forward Primer: AAACCTGGCTTGCTGATG<br>Reverse Primer: GGGGGCTAATGTTCTTGTCA
SOD2	Forward Primer: CTGGACAAACCTCAGCCCTAAC<br>Reverse Primer: AACCTGAGCCTTGGACACCAAC
CAT	Forward Primer: GACAATCAGGGTGGTGCTCC<br>Reverse Primer: GAATCGCATTCTTAGGCTTCTC

TRX2	Forward Primer: GTCCACACCACTGTGCGTGG<br>Reverse Primer: TTGCAGGGAGATGGCTCAGCG
TXNRD2	Forward Primer: GCACCTTTGACACCGTCCTGTG<br>Reverse Primer: CACCAGGATCTTCTGAGTGTCG
Prx5	Forward Primer: CCAATCAAGGTGGGAGATGCC<br>Reverse Primer: GCAGGTGTGTCTTGGAACATC
Prx3	Forward Primer: CCTTTGGATTTCACCTTTGTGTG<br>Reverse Primer: CAAACCACCATTCTTTCTTGGTG
COX IV	Forward Primer: ACGAGCTCATGAAAGTGTTGTG<br>Reverse Primer: AATGCGATACAACTCGACTTTCTC
ACTB	Forward Primer: TCATGAAGTGTGACGTGGACATC<br>Reverse Primer: CAGGAGGAGCAATGATCTTGATCT

1.2 Supplementary Table S2: Differentially-expressed molecules in RNA-sequencing data

2. Supplementary Figure

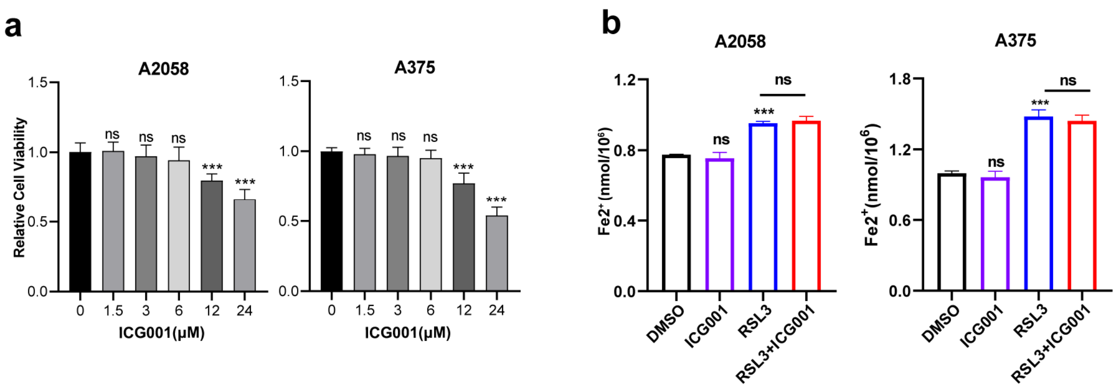

Supplementary Figure S1. (**a**) Relative cell viability of A2058 and A375 cell lines after the treatment with different concentrations of ICG001. (**b**) The intracellular level of Fe^{2+} in both A2058 and A375 cell lines after indicated treatment. Data represent the mean ± SEM of triplicates. *P* value was calculated by two-tailed Student's *t*-test. $^{***}P < 0.001$. ns, non-significant.
